# A Rapid PCR-Free Next-Generation Sequencing Method for the Detection of Copy Number Variations in Prenatal Samples

**DOI:** 10.3390/life11020098

**Published:** 2021-01-28

**Authors:** Xiya Zhou, Xiangbin Chen, Yulin Jiang, Qingwei Qi, Na Hao, Chengkun Liu, Mengnan Xu, David S. Cram, Juntao Liu

**Affiliations:** 1 Department of Obstetrics and Gynecology, Chinese Academy of Medical Sciences, Peking Union Medical College, Peking Union Medical College Hospital, Beijing 100730, China; zhouxiya@pumch.cn (X.Z.); jiangyl@pumch.cn (Y.J.); qiqingwei@pumch.cn (Q.Q.); haona@pumch.cn (N.H.); 2 Berry Genomics Corporation, Beijing 102200, China; chenxiangbin157@berrygenomics.com (X.C.); liuchengkun829@berrygenomics.com (C.L.); xumengnan001@berrygenomics.com (M.X.)

**Keywords:** chromosome disorders, copy number variation (CNV), PCR-free libraries, rapid copy number variation sequencing (rCNV-seq)

## Abstract

Next-generation sequencing (NGS) is emerging as a new method for the detection of clinically significant copy number variants (CNVs). In this study, we developed and validated rapid CNV-sequencing (rCNV-seq) for clinical application in prenatal diagnosis. Low-pass whole-genome sequencing was performed on PCR libraries prepared from amniocyte genomic DNA. From 10–40 ng of input DNA, PCR-free libraries consistently produced sequencing data with high unique read mapping ratios, low read redundancy, low coefficient of variation for all chromosomes and high genomic coverage. In validation studies, reliable and accurate CNV detection using PCR-free-based rCNV-seq was demonstrated for a range of common trisomies and sex chromosome aneuploidies as well as microdeletion and duplication syndromes. In reproducibility studies, CNV copy number and genomic intervals closely matched those defined by chromosome microarray analysis. Clinical testing of genomic DNA samples from 217 women referred for prenatal diagnosis identified eight samples (3.7%) with known chromosome disorders. We conclude that PCR-free-based rCNV-seq is a sensitive, specific, reproducible and efficient method that can be used in any NGS-based diagnostic laboratory for detection of clinically significant CNVs.

## 1. Introduction

There are over 100 recurrent chromosome disorders that affect the human population [1]. These syndromes are caused by copy number variations (CNVs) that include both whole chromosome and segmental aneuploidies that arise during gametogenesis or in the preimplantation period of embryo development [2]. While the large majority of fetal CNVs lead to early miscarriage [3,4], a small proportion are developmentally competent and are compatible with a livebirth outcome. Approximately 15% of all congenital abnormalities are associated with a pathogenic CNV [5]. 

The most common whole chromosome aneuploidies of the newborn are trisomies T21 (Down’s syndrome), T18 (Edward’s syndrome) and T13 (Patau’s syndrome) and the sex chromosome aneuploidies 45,XO (Turner’s syndrome), 47,XXX (Triple X syndrome), 47,XXY (Klinefelter’s syndromes) and 47,XYY (Jacob’s syndrome) [6]. On the other hand, segmental aneuploidies are associated with a variety of microdeletion and microduplication syndromes (MMS). While the disease prevalence of different MMS varies from around 1 in 3000 to 1 in 25,000 [7], as a collective group, they are relatively common with 22q11.2 microdeletions (DiGeorge Syndrome) being the most frequent MMS seen in the newborn [8]. 

In prenatal diagnosis practice, women with a high fetal risk for a chromosome abnormality are usually referred for invasive testing by amniocentesis at 15–16 weeks gestation or by chorionic villous sampling at 10–11 weeks gestation. High risk is generally inferred by either an abnormal maternal serum screening score, advanced maternal age or presence of a soft ultrasound marker or an ultrasound structural abnormality [5,9]. Invasive chromosome testing for aneuploidy is usually routinely performed by karyotyping, which has a CNV resolution of around 5–10 Mb in size [5]. For identification of smaller CNVs, chromosome microarray analysis (CMA) using high density SNP arrays is the most widely used method [10,11]. For both karyotyping and CMA, amniocytes are normally cultured to generate sufficient cells for analysis, and results are generally available after 2 weeks. More recently, next-generation sequencing (NGS) has been developed as an alternative method for detection of CNVs, with a detection resolution down to 0.1 Mb [12,13,14], which is sufficient to detect the vast majority of MMS. In a recent large cohort study of patients referred for invasive testing, we demonstrated that CNV-seq applied to amniocyte DNA samples can provide an increased yield of pathogenic CNVs compared to karyotyping [15]. More recently, in a study of over 1000 women referred for invasive testing, low-pass whole-genome sequencing performed similarly to CMA for detection of clinically significant CNVs [16]. 

In the field of molecular diagnostics, CMA is still the preferred methodology for identification of clinically significant CNVs in prenatal samples. To advance the clinical application of NGS, improvements in the sensitivity, specificity, reproducibility and versatility of NGS-based CNV detection methods are still needed before this technology can be generally considered for routine chromosome testing. One essential part of the NGS workflow is the library preparation method, with most CNV-seq tests currently using a low DNA input (50–200 ng) and a PCR step to amplify genomic fragments. As an alternative approach, we developed and validated a PCR-free-based rapid CNV-seq (rCNV-seq) method suitable for analysis of low nanograms amounts of genomic DNA from uncultured amniocytes. In a prospective study of 217 patients referred for chromosome testing, we show that rCNV-seq of the amniotic cell genomic DNA can reliably and accurately detect clinically significant CNVs.

## 2. Materials and Methods 

### 2.1. Patient Samples

All blood and amniocentesis samples were collected at the Prenatal Care Unit of Peking Union Medical College Hospital (PUMCH). The research study was approved by the Ethics Committee for Drug Clinical Trials in PUMCH (approval number KS2019136), and written informed consent was obtained from each patient. For validation studies, peripheral blood samples (2 mL) were taken from patients with known chromosome disorders and genomic DNA (gDNA) extracted using AxyPrep Mag Tissue-Blood gDNA kit (Axygen, Corning NY, USA). For patients with a suspected chromosome abnormality due to indications such as advanced maternal age, an abnormal maternal serum screening result or a structural abnormality revealed by ultrasound, invasive testing was performed on amniocentesis samples (10 mL of amniotic fluid) obtained at 15–16 weeks gestation. The fluid was centrifuged, and gDNA from the amniocyte cell pellet was purified using the AxyPrep Mag Tissue-Blood gDNA kit. Prior to CNV testing, amniocyte DNA was checked for maternal cell contamination (MCC) using an STR-based semi quantitative PCR assay [17]; MCC levels of <5% were considered acceptable for clinical testing. 

### 2.2. Construction of NGS Libraries 

**Figure 1:**
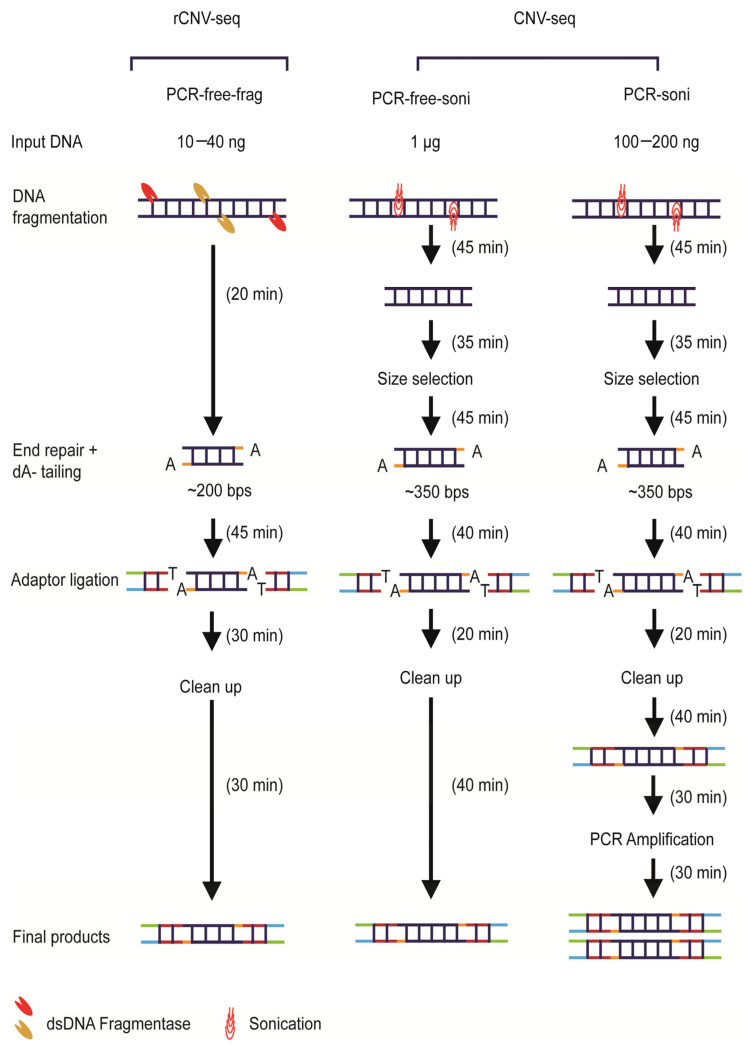
Overview of next-generation sequencing (NGS) library construction workflows. The overall time required to generate PCR-free-frag, PCR-free-soni and PCR-soni libraries was 2.1, 3.75 and 4.75 h, respectively.

An overview of the three library construction methods used in this study is shown in Figure 1. All genomic samples for library construction were quantified using Qubit 3.0 (Invitrogen, Waltham, MA, USA). For PCR-free-frag library construction used by rCNV-seq (Method 1), gDNA (10–40 ng) was initially treated by dsDNA fragmentase at 37 °C (NEBNext dsDNA Fragmentase, New England Biolabs, Ipswich, MA, USA) to produce smaller derivatives with an average size of ~200 bps. Prepared gDNA fragments were then end-repaired, A-tailed and then ligated with barcoded sequencing adaptors using a proprietary DNA repair kit (KR2000, Berry Genomics, Beijing, China) to generate libraries for sequencing. For PCR-free-soni (Method 2) and PCR-soni (Method 3) used for commercial CNV-seq, gDNA (1 µg, PCR-free-soni; 100–200 ng PCR-soni) was sheared by sonification and fragments of 350 bps size selected on agarose gels using TruSeq DNA PCR-Free Low Throughput Library Prep Kit (Illumina, San Diego, CA, USA) and TruSeq Nano DNA Low Throughput Library Prep Kit (Illumina, San Diego, CA, USA), respectively (Figure 1).

### 2.3. Copy Number Variation Sequencing and Data Analysis

Single end sequencing was performed on the NextSeq CN500 platform (Illumina, San Diego, CA, USA) with a run time of 6.5 h to generate approximately 5 million raw 45 bp reads per sample. Raw reads were then edited to remove artificial adaptor sequences, and the true 36 bp genome sequences were then mapped to the hg19 reference genome using the Burrows and Wheeler algorithm [18]. On average, approximately 2.8–3.2 million reads were uniquely mapped for data analysis. Reads were allocated to 20 kb bins along the length of each chromosome, and CNVs were identified from 24 chromosome copy number (CN) plots, as previously described [13,19]. Duplications were defined as CN >2.8, deletions CN <1.2, disomy (1.8 < CN < 2.2), mosaic trisomy (2.2 < CN < 2.8) and mosaic monosomy (1.2 < CN < 1.8). 

Genomic variant databases including DGV (Database of Genomic Variants), http://projects.tcag.ca/variation), OMIM (Online Mendelian Inheritance in Man) (http://www.omim.org), PubMed (http://www.ncbi.nlm.nih.gov/pubmed) and UCSC (University of California, Santa Cruz) (http://genome.ucsc.edu/, hg19) were used as a reference source of CNVs. The pathogenicity of detected CNVs was assessed following ACMG guidelines [20]. 

### 2.4. Karyotyping

Cultured amniocytes were karyotyped by standard procedures [21]. Cytogenetic analysis of Giemsa-stained metaphase spreads was performed at a resolution of 320 bands. 

### 2.5. Chromosome Microarray Analysis

CMA was performed using the CytoScan™ HD Array Kit (ThermoFisher Scientific, Waltham, MA, USA) according to the recommended protocol. The array contains more than 2.6 million SNPs and can detect copy number changes across the genome at a resolution of 25–50 kb. Genomic DNA samples were labeled and hybridized to the array according to the manufacturer’s recommended protocol. Fluorescence signals were scanned using the GeneChip scanner (ThermoFisher Scientific, Waltham, MA, USA) and chromosome copy number changes called by Applied Biosystems™ GeneChip Command Console Software (version 3.2.2).

## 3. Results

### 3.1. High Performance of PCR-Free-Based rCNV-Seq

**Figure 2:**
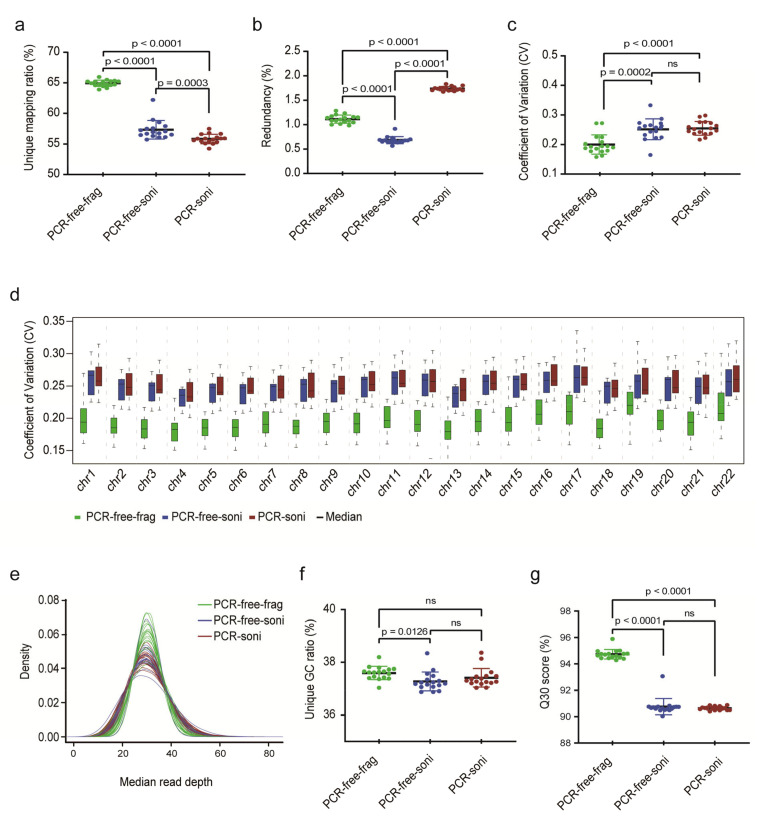
Comparison of key sequencing QC data to evaluate the performance of NGS library construction methods. (**a**) Ratio of unique read mapping. (**b**) Ratio of read redundancy. (**c**) Coefficient of variation (CV) (SD/mean) across the whole genome. (**d**) CV for each chromosome. (**e**) The uniformity of genome sequence coverage measured by median read depth. (**f**) Unique GC ratio of sequencing reads. (**g**) Q30 (chance that the base call is incorrect is 1 in 1000).

The performance of PCR-free-frag-based libraries (rCNV-seq) and two control PCR-free-soni and PCR-soni libraries (CNV-seq) (Figure 1) was bioinformatically assessed for 17 replicate normal female gDNA samples extracted from postnatal blood samples (Figure 2). The percentage of uniquely mapped reads was significantly higher for PCR-free-frag (rCNV-seq) compared to PCR-free-soni (*p* < 0.0001) and PCR-soni (*p* < 0.0001) libraries. Both PCR-free-frag and PCR-free-soni libraries had a significantly lower read redundancy rate compared to the PCR-soni libraries (*p* < 0.0001). The coefficient of variation (CV) achieved with PCR-free-frag libraries was also lower across all chromosomes and for each individual chromosome, compared to PCR-free-soni and PCR-soni libraries. The median read sequencing depth, indicative of increased bin read numbers, was also higher for PCR-free-frag compared to PCR-free-soni and PCR-soni libraries. Further, the Guanidine/Cytosine (GC) content of the sequencing reads was higher for PCR-free-frag than PCR-free-soni. Lastly, the Q30 values were significantly higher for PCR-free-frag compared to PCR-free-soni (*p* < 0.0001) and PCR-soni (*p* < 0.0001). Based on evaluation of these key Quality Control (QC) sequencing indicators, rCNV-seq using PCR-free-frag libraries showed the highest performance values.

**Figure 3:**
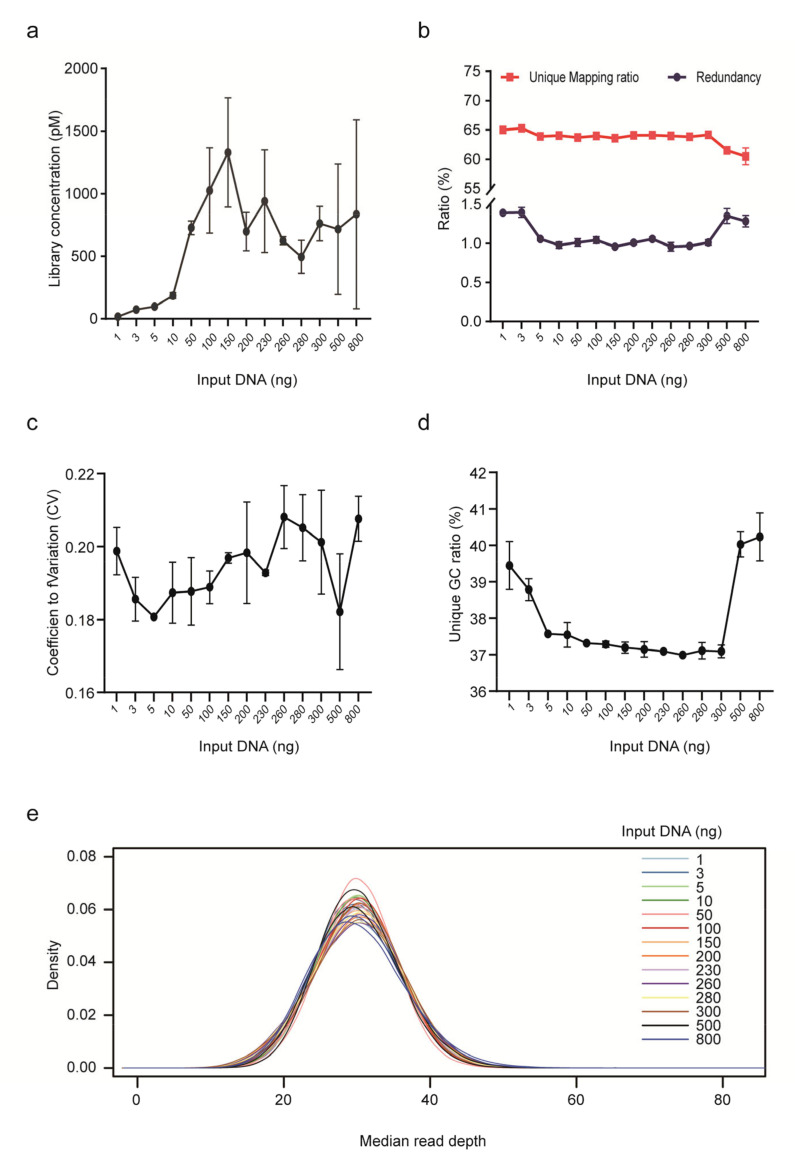
Performance of rCNV sequencing using different amounts of DNA inputs. (**a**) Library yield. (**b**) Ratio of unique read mapping and read redundancy. (**c**) CV. (**d**) Unique GC ratio. (**e**) Median read depth.

We further assessed the impact of input gDNA amount on library yield, unique mapping ratio and redundancy for PCR-free rCNV-seq (Figure 3). Mean DNA library concentrations were low (<200 p mole) at input DNA amounts <10 ng. Higher mean library yields suitable for sequencing were obtained with DNA input levels ranging from 50–800 ng. Despite different DNA inputs (1–800 ng), the unique mapping ratio was relatively stable at 60–65%. In terms of read redundancy, lower and higher DNA inputs were associated with a slightly higher ratio. Based on these assessment criteria, rCNV-seq provided high-quality sequencing data at input DNA amounts of ≥10 ng. 

### 3.2. Validation of rCNV-Seq Using PCR-Free-Frag Library Preparations 

**Figure 4:**
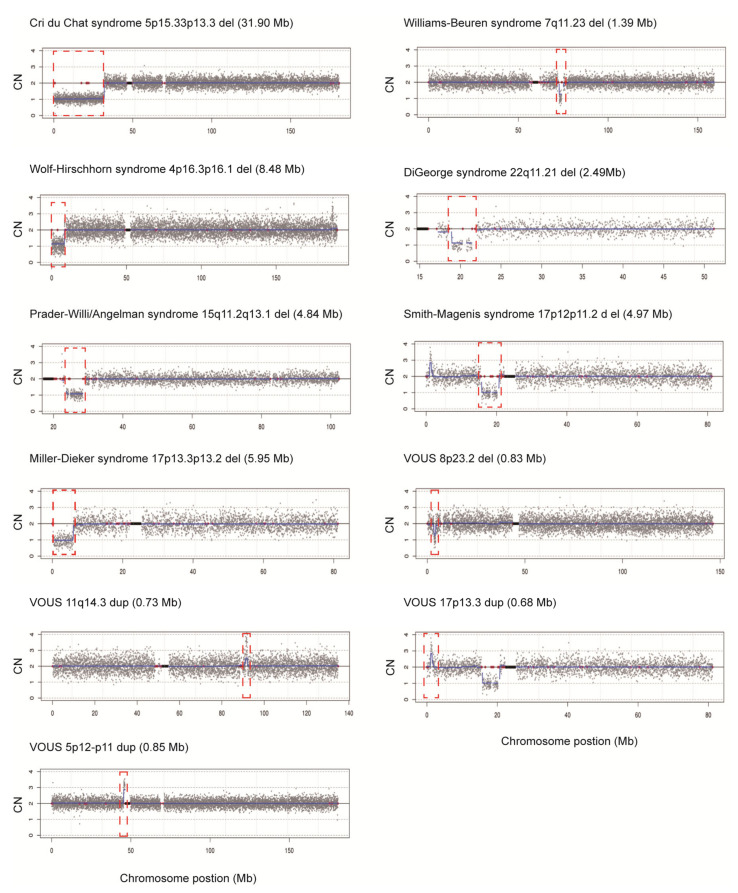
Chromosome plots produced from rCNV-seq analysis of samples with known CNVs. Y-axis, copy number and X-axis, copy number of the sequencing bins (gray dots). Blue line signifies the mean copy along the length of the chromosome. Red line indicates regions of repetitive DNA, and the black box denotes the centromere.

Our newly designed rCNV-seq method based on PCR-free-frag libraries was further evaluated for the ability to detect known chromosome disorders, previously detected by CMA. These included single samples identified with trisomies T21, T18 and T13 and sex chromosome aneuploidies (SCAs) 45,XO, 47,XXX, 47,XXY and 47,XYY. In addition, we also selected MMS samples including three cases each of Cri du Chat and William–Beuren syndrome, two cases each of Wolf–Hirschhorn and Di George syndrome and one case each of Prader-Willi/Angelman, Smith–Magenis and Miller–Dieker syndrome. Further, we also included four additional samples with variants of uncertain significance (VOUS) involving CNVS < 1 Mb in size. In these experiments, we used a low DNA input of 40 ng as the starting template for PCR-free-frag library construction. Following rCNV-seq analysis of all samples, the specific CNV that was originally identified by CMA was also detected by rCNV-Seq (Table 1, Figure 4). In addition, the CNV intervals defined closely matched those defined by CMA and the expected CN prediction of one for deletions and three for duplications was observed. Taken together, our rCNV-seq protocol was highly sensitive for detecting the underlying causative CNVs associated with these MMS. 

**Figure 5:**
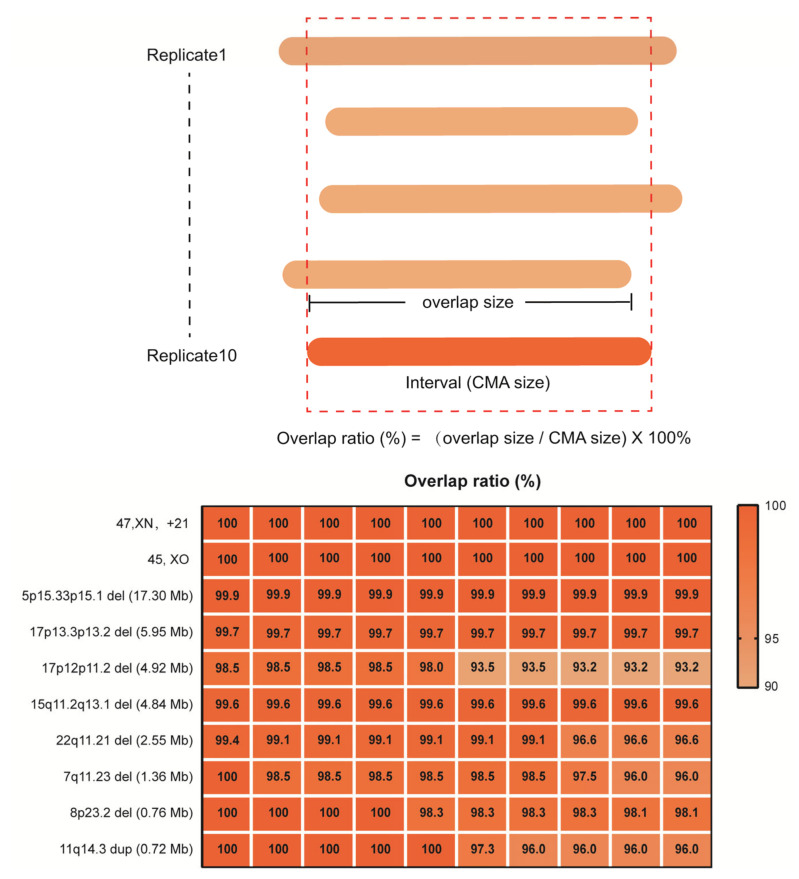
Reproducibility of rCNV-seq for detection of CNVs, copy number (CN) and genome interval coordinates. The top panel describes how the CNV intervals detected by rCNV-seq were compared to CMA results. The bottom panel provides the level of CNV concordance (%) between rCNV-seq and CMA.

In detailed reproducibility experiments using 10 replicate samples of 10ng for library construction, we further evaluated the reliability of rCNV-seq to correctly detect the precise CNV interval, benchmarking it against the CNV interval previously defined by CMA (Figure 5). For 45,XO, one copy of chromosome X was clearly deleted (CN = 1) in all replicates. Likewise, for T21, the whole q arm of chromosome 21 was clearly duplicated (CN = 3) in all replicates. For the 5p15.33p15.1 (17.3 Mb), 17p13.3p13.2 (6.0 Mb), 17p12p11.2 (4.9 Mb), 15q11.2q13 (4.8 Mb), 22q11.21 (2.6 Mb), 7q11.23 (1.36 Mb) and 8p23.2 (0.83 Mb) deletions and the 11q14.3 duplication (0.72 Mb) CNVs, all were detected in the 10 replicates and the mapped interval closely matched the coordinates of the CMA-defined interval (within 93–100%). 

### 3.3. Diagnostic Performance of rCNV-Seq Using Prenatal Samples 

**Figure 6:**
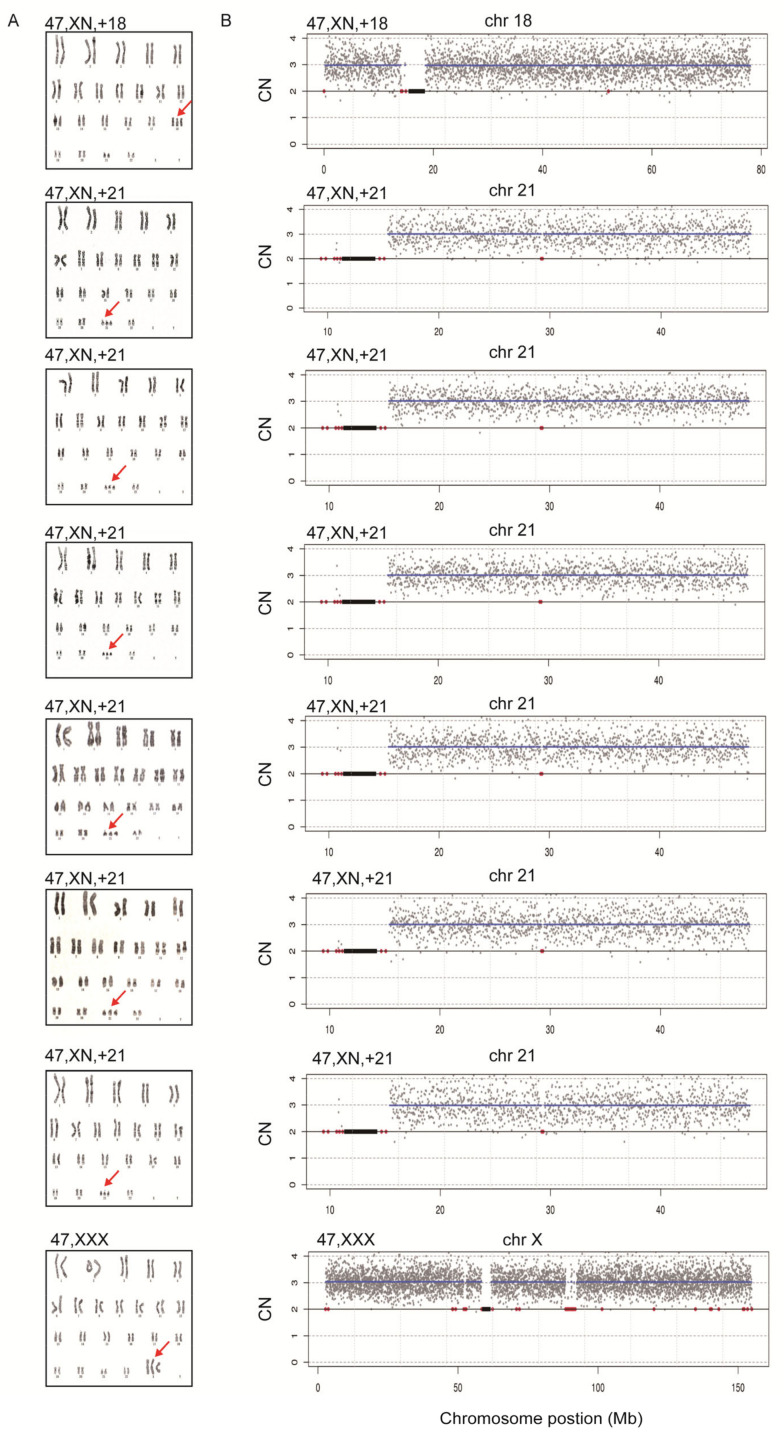
Confirmation of whole chromosome aneuploidies by karyotyping. A. Karyotyping. Red arrows indicate chromosome gains. B. rCNV-seq results. The chromosome copy number gains determined by rCNV-seq were concordant with karyotyping. rCNV-seq plots. Y-axis copy number and X-axis mean copy number of the sequencing bins (gray dots). Blue line signifies the mean copy along the length of the chromosome. Red line indicates regions of repetitive DNA, and the black box denotes the centromere.

**Figure 7:**
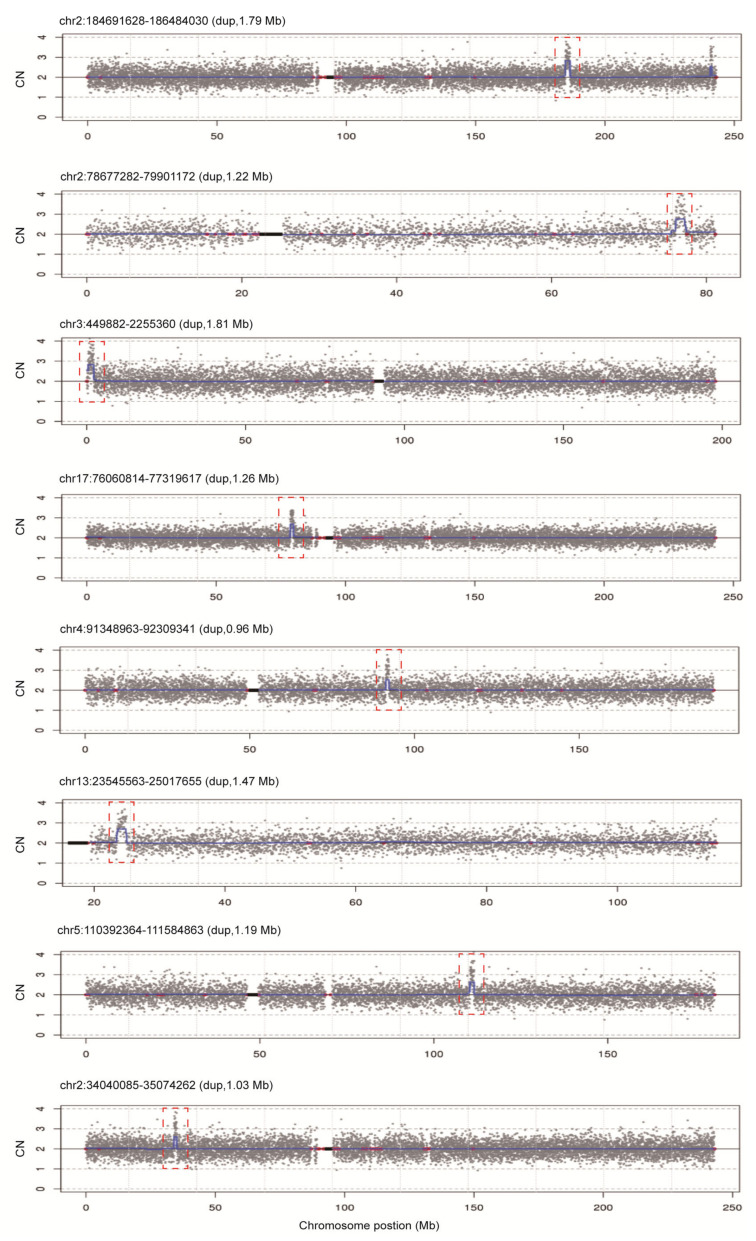
rCNV-seq detection of CNVs in 217 clinical samples. All CNVs identified were classified as VOUS. Y-axis copy number and X-axis mean copy number of the sequencing bins (gray dots). Blue line signifies the mean copy along the length of the chromosome. Red line indicates regions of repetitive DNA, and the black box denotes the centromere.

To assess the diagnostic performance of rCNV-seq, 40 ng of amniocyte gDNA was analyzed from 217 pregnant women referred for chromosome testing for a variety of different clinical indications (Table 2). MCC checks confirmed that all amniocyte genomic DNA samples were >99% fetal DNA. In eight samples, rCNV-seq detected whole chromosome fetal aneuploidies, including T21 (n = 6), T18 (n = 1) and 47,XXX (n = 1). These aneuploidies were confirmed by follow up karyotyping (Figure 6). There were no samples carrying pathogenic fetal CNVs associated with an MMS. However, eight samples were identified with small non-pathogenic duplications (0.96–1.81 Mb) that were classified as VOUS (Figure 7).

## 4. Discussion

In this study, we developed rCNV-seq based on a PCR library free sequencing methodology for detection of clinically significant CNVs in prenatal samples. The optimized rCNV-seq method accommodates input DNA sheared enzymatically without additional size selection steps and then combines the end repair and dA (adenine)-tailing into one step, removing the need for a PCR process and making library preparation much faster and easier (Figure 1). Equally important, library construction can be completed in a single tube, making the workflow more conducive to automation. In validation studies, rCNV-seq was highly sensitive and specific for the detection of common whole chromosome aneuploidies such as T21, T18, T13, 45,XO, 47,XXY, 47,XXX and 47,XYY as well as for segmental aneuploidies associated with different types of MMS. Detection of CNVs was highly reproducible, even when gDNA amounts as low as 10 ng were used to construct PCR libraries. In clinical studies of amniocentesis prenatal samples at risk for a fetal CNV, we also showed the ability of rCNV-seq to correctly identify both pathogenic and VOUS CNVs. 

One of the main drivers for reliable and accurate calling of CNVs is the quality and depth of the sequencing data that are binned across each chromosome. In this regard, rCNV-seq based on PCR-free libraries demonstrated improved performance over current CNV-seq methods, showing an average Q30 for the sequencing data as high as 94%. Compared to PCR, PCR-free-frag libraries achieved a higher proportion of uniquely mapped reads (fewer duplicate reads), a lower read redundancy and a smaller CV for all chromosomes, which are important parameters to improve the signal-to-noise ratio and allow more accurate detection of CNVs and their correct copy number. In addition, rCNV-seq exhibited some additional advantages for implementation of the method into molecular diagnostic laboratories with NGS capacity. Firstly, PCR-free libraries can be generated in around 2 h compared to 4–5 h for current PCR-free and PCR-dependent commercial methods. Thus, from sample receipt in the morning and with a sequencing run time of 6.5 h on the Next-Seq platform, it is possible to generate same day results and improves the overall efficiency of laboratory workflow and staff time. Second, the method can generate representative libraries from a wide range of input DNA amounts, improving versatility for the analysis of different sample types and compromised samples where the gDNA is limiting. 

Benchmarking against CMA, rCNV-seq demonstrated a high degree of accuracy and reproducibility. For the majority of CNVs tested, there was a high concordance between the two methods for correctly calling the CNV interval and copy number. There were some exceptions where the CNV interval varied up to 33% for the genome location called. These differences are probably related to the inherent limitations of both techniques, whereby CMA has reduced probe coverage for some genome positions, whereas rCNV-seq has reduced coverage in highly repetitive regions. While high sensitivity and specificity is an important parameter for calling CNVs, reproducibility is also a key factor for defining the resolution of CNV detection, since each rCNV-seq analysis is based on a set of randomly mapped sequencing reads mapped across the 24 chromosomes. Previous studies have shown that while CNVs as small as 0.1 Mb can be detected by CNV-seq [13], there were no data provided on the reproducibility of detection. In this study, using gDNA inputs of 10 ng, high reproducibility of CNV detection was demonstrated for CNVs as large as 31.9 Mb to as small as 0.7 Mb. Nonetheless, further studies on a range of smaller CNVs are still needed to determine the minimum resolution of rCNV-seq for a single-pass diagnostic test. 

Based on our findings, rCNV-seq is also suitable as an alternative to CMA for routine prenatal testing where a gDNA sample is generally available from cultured amniocytes. However, there are also clinical situations whereby rCNV-seq has some clear advantages over CMA. Firstly, some women are referred for an invasive chromosome test close to the legal limit for termination of pregnancy, which is usually 20 weeks in most countries [22]. Secondly, the amniocentesis samples collected can be substandard, lacking sufficient fetal cells for analysis and, occasionally, there can be cell culture failure [23]. In these two scenarios, it may not be possible to use CMA methods, because they rely on cultured amniocytes (one to two weeks) and a minimum of 0.5–1 µg of input DNA for analysis. In these urgent clinical situations, it will be possible to use rCNV-seq to achieve a rapid and accurate diagnosis using either low amounts of gDNA from uncultured amniocytes or even the available cell free fetal DNA, which has been previously shown to be an ideal NGS template for CNV detection in pregnancies with abnormal fetal ultrasound structural abnormalities [24]. 

## 5. Conclusions

PCR-free-based rCNV-seq is a rapid, sensitive, specific and efficient method that can be used in any NGS-based diagnostic laboratory to achieve a reliable and accurate CNV diagnosis. Further studies on a large number of amniocentesis samples are needed to define the true clinical utility and versatility of rCNV-seq for prenatal chromosome testing.

## Figures and Tables

**Table 1:** Validation of rCNV-seq for detection of known CNVs.

Chromosome Disorders	CMA	rCNV-Seq (PCR-Free-Frag)	Interpretation
**Common trisomies**			
T21 ^ǂ^	47XN,+21	47XN,+21	Concordant
T18	47XN,+18	47XN,+18	Concordant
T13 ^ǂ^	47XN,+13	47XN,+13	Concordant
**Sex chromosome aneuploidies (SCAs)**			
45,XO ^ǂ^	45,XO	45,XO	Concordant
47,XXY ^ǂ^	47,XXY	47,XXY	Concordant
47,XXX	47,XXX	47,XXX	Concordant
47,XYY	47,XYY	47,XYY	Concordant
**Microdeletion/microduplication syndromes (MMS)**			
**Cri du Chat syndrome (5p15.3-p15.2)**			
Sample 1 ^ǂ^	5p15.33p13.3(113576-31928290) ×1, 31.81 Mb	5p15.33-p13.3(del, 31.90 Mb)	Concordant
Sample 2	5p15.2p15.33(151,737-14,756,030) ×1, 14.6 Mb	5p15.33-p15.2(del, 17.30 Mb)	Concordant
Sample 3	5p13.3p15.33(151,737-33,120,547) ×1, 32.97 Mb	5p15.33-p13.3(del, 33.14 Mb)	Concordant
**Williams–Beuren syndrome (7q11.23)**			
Sample 1 ^ǂ^	7q11.23(72653992-74146927) ×1, 1.49 Mb	7q11.23(del, 1.32 Mb)	Concordant
Sample 2 ^ǂ^	7q11.23(72549979-74374748) ×1, 1.82 Mb	7q11.23(del, 1.36 Mb)	Concordant
Sample 3	7q11.23(72,726,578-74,139,390) ×1, 1.41 Mb	7q11.23(del, 1.39 Mb)	Concordant
**Wolf–Hirschhorn syndrome (4p16.3)**			
Sample 1 ^ǂ^	4p16.1p16.3(71,552-8,510,870) ×1, 8.44 Mb	4p16.3-p16.1(del, 8.48 Mb)	Concordant
Sample 2	4p16.1p16.3(143,413-8,368,180) ×1, 8.22 Mb	4p16.3-p16.1(del, 8.35 Mb)	Concordant
**DiGeorge syndrome (22q11.2)**			
Sample 1 ^ǂ^	22q11.21(18648855-21800471) ×1, 3.15 Mb	22q11.21(del, 2.49 Mb)	Concordant
Sample 2	22q11.21(18,919,942-21,440,514) ×1, 2.52 Mb	22q11.21(del, 2.55 Mb)	Concordant
**Prader-Willi/Angelman syndrome (15q11-q13)**			
Sample 1 ^ǂ^	15q11.2q13.1(23290787-28560664) ×1, 5.27 Mb	15q11.2-q13.1(del, 4.84 Mb)	Concordant
**Smith–Magenis syndrome (17p11.2)**			
Sample 1	17p11.2p12(15,816,892-20,193,169) ×1, 4.38 Mb	17p12-p11.2(del, 4.92 Mb)	Concordant
**Miller–Dieker syndrome (17p13.3-p13.2)**			
Sample 1 ^ǂ^	17p13.3p13.2(1,972,209-5,945,876) ×1, 4.0 Mb	17p13.3p13.2(del, 5.95 Mb)	Concordant
**VOUS (8p23.2)**			
Sample 1 ^ǂ^	8p23.2(3,427,306-4,275,392) ×1, 0.8 Mb	8p23.2(del, 0.83 Mb)	Concordant
**VOUS (11q14.3)**			
Sample 1	11q14.3(91,347,506-92,085,142) ×3, 0.7 Mb	11q14.3(dup, 0.73 Mb)	Concordant
**VOUS (17p13.3)**			
Sample 1	17p13.3(1,004,599-1,518,383) ×3, 0.51 Mb	17p13.3(dup,0.68 Mb)	Concordant
**VOUS (5p12-p11)**			
Sample 1 ^ǂ^	5p12p11(45,455,695-46,242,541) ×3, 0.78 Mb	5p12-p11(dup, 0.85 Mb)	Concordant

^ǂ^ Amniotic fluid.

**Table 2:** Clinical performance of rCNV-seq for detection of CNVs in 217 amniotic fluid samples.

Primary Indication for Chromosome Testing	Sample (n)	rCNV-Seq Result	
		Abnormal	Normal
**Advanced Maternal Age (≥35 Years)**	152	4 (T21)	148
Abnormal serum screening result (<35 years)	41	2 (T21 and 47,XXX)	39
Previously given birth to child with a chromosomal disorder	8	1 (T18)	7
Structural abnormality by ultrasound	7	1 (T21)	6
One parent is a carrier of a chromosomal disorder	2	0	2
Advanced maternal age (≥35 years) and secondary indication ^#^	7	0	7
Total	217	8	209

^#^ Secondary indications included abnormal serum screening result, previously given birth to child with a chromosomal disorder, structural abnormality by ultrasound or one parent is a carrier of a chromosomal disorder.

## Data Availability

The data presented in this study are available on request from the corresponding author.
